# CRISPR/Cas9-mediated t(4;11) translocation in human hematopoietic stem/precursor cells demonstrates plasticity to differentiate into either the myeloid or lymphoid lineage

**DOI:** 10.1038/s41375-025-02791-4

**Published:** 2025-10-27

**Authors:** T. Benz, P. Larghero, C. Meyer, T. Hanewald, D. Brüggmann, A.-E. Hentrich, F. Louwen, R. Marschalek

**Affiliations:** 1 Institute Pharm. Biology, Biocenter, Goethe-University, Frankfurt/Main, Main Germany; 2 Department Obstetrics and Prenatal Medicine, Goethe-University, Frankfurt/Main, Main Germany

**Keywords:** Acute lymphocytic leukaemia, Cancer models

## Abstract

The chromosomal translocation t(4;11)(q21;q23) is frequently diagnosed in *KMT2A*-r Acute Leukemia patients. Although we understand much about the function of both wildtype KMT2A and AFF1 multiprotein complexes, little is known about the molecular actions the two fusion proteins KMT2A::AFF1 and AFF1::KMT2A during the very early steps of disease onset and progression. Most published data have been generated in t(4;11) cell lines or transplanted mouse models, where exactly this process remains a black box. Here, we present the results of our efforts to establish a t(4;11) chromosomal translocation in human hematopoietic stem/precursor cells by CRISPR/Cas9. These genetically modified cells can be expanded over 5-6 months in vitro and their potential to differentiate was examined with IL-7 supplementation. The benefit of this model system is that (1) both reciprocal fusion proteins are concomitantly present, and (2) a molecular surveillance is possible at any timepoint through analysis of RNA, DNA or protein. Thus, the CRISPR/Cas9 technique allowed us to create a bona fide model system to study the very early steps of leukemia onset at the molecular level. In conclusion, this approach is the fastest way to investigate and characterize *KMT2A*-r fusions in primary human cells.

## Introduction

The *KMT2A/MLL* gene has been described by 4 different groups in 1991 and 1992 to be the gene located at 11q23 which was involved in chromosomal translocations of acute leukemia patients [1–7]. Over the last decades, the *KMT2A* recombinome has been unraveled and currently exhibits 107 direct *KMT2A* fusions (*KMT2A::X*), 16 out-of-frame fusions to partner genes, and 16 reciprocal *KMT2A* fusions (*X::KMT2A*), all associated with the development of acute leukemia in patients [8].

Early experiments using retroviral transduction of murine hematopoietic stem/progenitor (LSK) cells, and their subsequent transplantation into C57BL/6 mice demonstrated that the expression of distinct *KMT2A* fusion genes was necessary and sufficient to cause acute leukemia without any other additional mutation [9–11]. Leukemia onset was usually observed within 6-9 months of latency in mouse model systems, indicating that the conversion process from a normal cell to a pre-leukemic state and to a full-blown leukemia takes time.

In subsequent years, different molecular functions of the KMT2A protein were elucidated. The KMT2A protein forms a multi-protein complex and is recruited via the N-terminally bound MEN1/LEDGF to transcription factors present at gene promoters in order to imprint the chromatin with H3K4_me3_ and other signatures [12–14]. This ensures a stable transcription of target genes and prevents the counteracting activity of either the SUV39H1/2/HP1 complex (H3K9_me3_) or the polycomb repressor complex II (H3K27_me3_); these signatures are subsequently recognized by polycomb repressor complex I to mediate the ubiquitinylation of H2AK119 [15]. Thus, the functional disruption of the KMT2A protein complex by any of the above described genetic aberrations causes a massive deregulation of gene transcription via the direct KMT2A::X fusion protein, while the reciprocal X::KMT2A fusion protein exhibits an important chromatin opening function which is necessary for the leukemogenic activity of the corresponding direct fusion protein [8, 16, 17].

These extreme changes observed in cells that acquired a genetic rearrangement of the *KMT2A* gene is then translated into cellular reprogramming, accompanied by the appearance of a specific genetic program which was first observed when *KMT2A*-rearranged patients were analyzed for their differential gene expression [18, 19]. These gene signatures typically exhibit either upregulated *HOXA* genes, as well as the upregulation of *MEIS1* and/or *PBX* family members, or upregulated IRX1 or IRX2 [20]. The latter program confers a more severe outcome and a higher rate of relapses [21, 22] which is due to the overexpression of EGR3 and the checkpoint inhibitor ICOSLG [23, 24].

However, a missing link in our understanding is the timepoint of conversion, namely, to understand the precise timing and the molecular consequences when a normal hematopoietic stem/precursor cell is converted into a preleukemic cell, and finally into leukemia initiating cells (LICs). All efforts to investigate these early timepoints ex vivo have failed so far.

A solution to this problem was the CRISPR/Cas9 technology which allows to establish chromosomal translocations in human hematopoietic stem/precursor cells either deriving from fetal liver, umbilical cord blood or adult bone marrow [25–30]. We applied our recently published protocol for the generation of CRISPR/Cas9-mediated *KMT2A* chromosomal translocations [31] which allows a rapid outgrowth of the genetically modified cells in vitro, and subsequently, to study these *KMT2A*-translocated cells during their new commitment by flow cytometry and molecular profiling.

Here, we describe the results of an experiment that traced an induced t(4;11) chromosomal translocation over 5-6 months, where we monitored clonality, surface expression of distinct CD markers, and performed gene expression profiling. The results demonstrate that the in vitro suspension culture exhibits early markers of leukemia after only 4-5 weeks, while cells still exhibit sufficient plasticity upon stimulation with a single additional cytokine, IL-7, to drive their lineage commitment into the lymphoid direction between days 40-70. The final gene signatures are highly similar to that one’s deriving from t(4;11) leukemia patients. The implications to grow such *KMT2A*-translocated cells ex vivo and to monitor the conversion process from normal hematopoietic cells into pre- or even leukemic cells by molecular methods will be discussed.

## Material and methods

### Umbilical cord blood samples

The use of pseudonymized umbilical cord blood (UCB) samples as well as the declaration of consent from the parents of the umbilical cord blood donors were assessed and formally approved by the Ethics Committee of the Goethe University (approval# 2021-499 from 20.01.2022). The umbilical cord and placenta blood were collected in cord blood collection bags directly after cesarean section or natural birth. The CD34^+^ hematopoietic stem and progenitor cells (HSPCs) were isolated from the umbilical cord and placenta blood via magnetic activated cell sorting (MACS) and directly used for suspension cell culture.

### CD34^+^ HSPC purification from UCB

CD34^+^ HSPC purification was carried out using a previously described protocol [31]. The CD34^+^ HSPCs were freshly purified from whole blood of the umbilical cord and placenta, collected in cord blood collection bags (Macopharma; #MSC1200PU) after birth [31]. (The collected cord blood can be stored at 4°C for 3 days). A buffy coat centrifugation 800 x g for 10 min (acceleration 9/deceleration 0) was performed of the whole blood sample, separating the plasma from the buffy coat layer and the erythrocytes. The plasma was sterile filtered (0.2 µm filter) twice and used for suspension cell culture. Density gradient (Pancoll (1.077 g/ml); #P04-60500) centrifugation 400 x g for 35 min (accerlation 1/deceleration 0) was performed to isolate cord blood mononuclear cells (CBMCs). CD34^+^ HSPCs were isolated via MACS (Miltenyi; #130-100-453) and cultured in StemSpan SFEMII (Stemcell Technologies; #09655) supplied with 10% donor plasma, 1% Penicillin/Streptomycin (Capricorn Scientific; # PS-B), stem cell factor (SCF) (100 ng/ml) (ThermoFisher Scientific (former Peprotech); #HHSC6), FLT3-ligand (FLT3-L) (100 ng/ml) (ThermoFisher Scientific (former Peprotech); #HHSC6) and thrombopoietin (TPO) (100 ng/ml) (ThermoFisher Scientific (former Peprotech); #HHSC6) for 48 h. On day 40, cells were treated with additional IL-7 (100 ng/ml) (Biolegend; #581906).

Optional: If necessary, the cells can be cryopreserved. 1 ×10^6^ to 5 ×10^6^ cells were pelleted at 300 x g for 5 min, the supernatant was discarded, and the cell pellet was resuspended in 1 ml 90% FBS (Capricorn Scientific; #FBS-11A) and 10% DMSO (Carl Roth; #A994.2). The cells were transferred in cryovials and frozen in Mr. Frosty™ Freezing Containers (ThermoFisher Scientific; #5100-0001) at -80°C.

### CRISPR/Cas9-editing and transfection

The CRISPR/Cas9-editing and transfection of CD34^+^ UCB HSPCs were carried out using a previously described protocol [31]. The *KMT2A/MLL* and *AFF1/AF4* sgRNAs were designed based on in house patient NGS breakpoint data [8]. The programs CHOPCHOP and CCTop were used for sgRNA design. In vitro transcribed sgRNAs were first tested in an in vitro Cas9 cleavage assay to determine sgRNA editing efficiency and in the K562 test cell line to test the capability of the sgRNAs to induce the t(4;11) chromosomal translocation. After that, the best suitable sgRNAs (see Table 1) were ordered as chemically modified synthetic sgRNAs (Synthego) [32, 33]. The CRISPR/Cas9-editing and transfection of the CD34^+^ UCB HSPCs was performed on day 2 of culture after the blood purification. For the electroporation, 0.5 ×10^6^ to 1.5 ×10^6^ CD34^+^ UCB HSPCs were harvested and electroporated with Cas9-sgRNA ribonucleoproteins (RNPs). The RNPs for *KMT2A* intron 9 and *AFF1* intron 3 were generated by pre-incubating 7.5 µM sgRNA and 3.75 µM Cas9 protein (PNA Bio; #CP02) separately at 37°C for 15 min followed by transfection into the cells with the Lonza 4D program CA-137. *KMT2A/MLL* intron 9: Ensemble nomenclature: ENSG00000118058; Transcript: *KMT2A*-234 ENST00000710560.1. *AFF1/AF4* intron 3: Ensemble nomenclature: ENSG00000172493; Transcript: *AFF1*-201 ENST00000307808.10. The cells were cultured in fresh stem cell medium supplied with 60 µM z-vad-fmk (Caspase-inhibitor; Enzo Life Science; #ALX-260-020-M005).

**Table 1:** sgRNA sequences and oligonucleotides.

sgRNAs:		
Name	Sequence 5’ → 3’	Source
sgMLL.intron 9	U*U*C*CUAGGCUAGAACAUGUG + 80-mer Synthego modified EZ Scaffold	Synthego
sgAF4.intron 3	C*A*G*UGUGGCGUGUAUUACGU + 80-mer Synthego modified EZ Scaffold	Synthego

Oligonucleotides:		
Name	Sequence 5’ → 3’	Source
MLL.ex9.sg.gDNA fw	CAAAAACCAAAAGAAAAGGTGAGGAGA	Eurofins
MLL.ex10.gDNA rev	ATTGACCGGAGGTGGTTTTTCCTAAG	Eurofins
AF4.I3.sg.1 gDNA fw	CTTATTTCTTCCTCAAAACAACCCTGTAAG	Eurofins
AF4.I3.sg.1 gDNA rev	GGGGAAGGAATAGGGGGAAGAT	Eurofins
MLL 8.3	CCCAAAACCACTCCTAGTGAG	Eurofins
MLL 13.5	CAGGGTGATAGCTGTTTCGG	Eurofins
AF4.3	GTTGCAATGCAGCAGAAGCC	Eurofins
AF4.5	ACTGTCACTGTCCTCACTGTC	Eurofins

### RNA and DNA isolation

On day 3 of culture post blood purification or after 24 h post-transfection, RNA and genomic DNA (gDNA) were isolated from the CRISPR/Cas9-edited CD34^+^ UCB HSPCs. The isolation of RNA and gDNA was performed with the AllPrep DNA/RNA micro kit (Quiagen; #80204). The RNA was transcribed into complementary DNA (cDNA) with the SuperScript II reverse transcriptase (Invitrogen; #18064014) to analyze the *KMT2A/MLL::AFF1/AF4* and *AFF1/AF4:: KMT2A/MLL* fusion transcripts. The gDNA was used to analyze the presence of different chromosomal fusions and to determine the primary sequence of NHEJ-repaired breakpoints after the CRISPR/Cas9-induced dsDNA strand breaks.

### Western Blot

For cell lysis, 1 ×10^6^ cells were resuspended in a 2 x sample reducing buffer containing DTT (100 mM), sodium dodecyl sulphate (SDS) (4%), glycerol (20% v/v), bromophenol blue (1% v/v), and TRIS, pH 6.8 (125 mM). The lysis sample was boiled on a heat block at 100°C for 10 min. During the lysis process the sample was occasionally vortexed. The lysate was centrifuged at 13,200 rpm at 4°C for 15 min to pellet cell debris and the supernatant was transferred into a fresh 1.5 ml Eppendorf tube and stored at -20°C for short term or -80°C for long term storage if not used immediately. 15 µl of the sample were loaded on a 7% Bis-Tris-polyacrylamide gel. The protein was blotted on a nitrocellulose membrane (0.2 µM; Bio-Rad; #1620112) via tank blotting system at 4°C, 30 V for 17 h. over night. The membrane was blocked in PBS 0.2% Tween20 and 5% nonfat milk. The following antibodies were used: anti-MLL/HRX (N-Terminus; clone N4.4; Sigma Aldrich; #05-764) and anti-Vinculin (clone EPR8185; Abcam; #ab129002). The membrane was imaged via the ChemiDoc XRS+ System (Biorad).

### Genomic profiling of subclones

Different clones appeared on the genomic DNA level after the induction of the chromosomal translocation. As a result of non-homologous end joining (NHEJ) the chromosomal repair of the double strand (ds)DNA breaks led to insertions of random filler DNA, deletions, or perfect fusions. The PCR products of the gDNA-breakpoint amplification were subcloned into the pCR2.1-TOPO TA-vector (Invitrogen; #450641) to separate the different clones and analyze each amplicon in detail via sanger sequencing. The sequences were aligned and analyzed with the CLC sequence viewer to unveil the oligoclonality of the bulk culture.

### Flow cytometry analyses

About 5 ×10^4^ to 5 ×10^5^ cells were transferred into a fresh 1.5 ml Eppendorf tube and centrifuged at 300 x g for 5 min. The cells were washed in 1 ml Dulbecco’s Phosphate Buffered Saline (DPBS), resuspended in 100 µl DPBS supplied with 0.5% bovine serum albumin (BSA) and stained with 1 μl of fluorophore-conjugated monoclonal antibodies at RT in the dark for 15 min. The cells were washed twice with 1 ml of DPBS 0.5% BSA and finally resuspended in 300 μl DPBS 0.5% BSA. For viability staining, 3 μl of 7-AAD were added and incubated 2 min before the measurement. The samples were measured with the BD FACSVerse™ instrument and analyzed with the FlowJo software. The following antibodies have been used: anti-human CD45-FITC (clone REA747; Miltenyi; #130-110-769), anti-human CD33-PE-Vio770 (clone REA775; Miltenyi; #130-111-138), anti-human CD123-APC-Vio770 (clone AC145; Miltenyi; #130-113-885), anti-human CD56-APC (clone REA196; Miltenyi; #130-113-872), anti-human CD19-BV510 (clone HIB19; BD Biosciences; #740164), anti-human CD20-V450 (clone L27; BD Biosciences; #561164), anti-human lineage cocktail 1(lin 1) [anti-human CD3-FITC (clone SK7), anti-human CD14-FITC (clone 3G8), anti-human CD16-FITC (clone SJ25C1), anti-human CD19-FITC (clone L27), anti-human CD20-FITC (clone MφP9), anti-human CD56-FITC (clone NCAM16.2)] (BD Biosciences; #340546), anti-human CD10-APC-Vio770 (clone 97C5; Miltenyi; #130-128-300), anti-human CD90-APC-Vio770 (clone REA879; Miltenyi; #130-114-905), anti-human CD45RA-BV-510 (clone HI100; BD Biosciences; #563031) and the viability stain 7-AAD (Miltenyi; #130-111-568).

### Cell trace proliferation assay

The CellTrace™ Violet Cell Proliferation Kit (Thermo Scientific; #C34557) was used to track cell proliferation and cell division. A 5 mM stock was prepared by adding 20 µl of DMSO to the cell trace dye. 1 ×10^6^ cells were pelleted at 300 x g for 5 min, the supernatant was discarded, and the pellet was resuspended in 1 ml DPBS supplied with 0.5% BSA. 1 µl of 5 mM CellTrace™ Violet dye was added to the cell suspension and incubated at 37°C in the dark for 20 min. The cells were washed twice in 9 ml DPBS and cultivated in the appropriate medium afterwards. After 3 h, a small aliquot of the cells was measured via flow cytometry with 7-AAD as viability marker (488 nm excitation, 647 nm emission) and CellTrace™ Violet dye (405 nm excitation, 445/450 nm emission).

### Fluorescence in situ hybridization (FISH)

The FISH experiments were performed according to the manufacturers protocol. The XL KMT2A BA break apart probe (#D-5090-100-OG) from MetaSystems was used for these experiments. DAPI/Antifade (MetaSystems; #-0902-500-DA) was used to detect the nucleus. The fluorescence microscopy was performed with the Axio Observer Z1 (Zeiss).

### Gene expression profiling

For the analysis of the gene expression data, principal component analysis (PCA) of all triplicates was performed as a quality control. Total RNA isolated at the indicated timepoints was subjected to MACE-SEQ analysis at GenXPro (https://genxpro.net; Frankfurt/Main, Germany). Differential gene expression analysis was performed at GenXPro by using the R program. The log2 FC was calculated from the non-CRISPR-edited day 14 vs. CRISPR-edited day 14, day 30, day 70 CD19^+^ and day 70 CD19^-^_._ For more detailed analyses, the tpm (transcripts per million) values of data derived from GenXPro were used for the calculation of the mean. In case of the appearance of the number 0 in a sample (log2 FC not calculable), 0.1 was added to both tpm means, the CRISPR-edited sample as well as the non-CRISPR-edited sample. The *p*-value was calculated with one-tailed paired student’s *t*-test from the triplicates of the CRISPR-edited sample and the triplicates of the non-CRISPR-edited samples. The *p* values were described as non-significant: p > 0.05 and significant: p ≤ 0.05. The Heatmaps were generated with the online tool Morpheus (Heatmap design). The Volcano plots were generated with the online tool VolcaNoseR. The Gene set enrichment analysis (GSEA) was performed with the online tool Enrichr. Gene overlap analysis was performed by using the recently published gene expression data from different hematopoietic subpopulations [34] and comparing them to our signatures.

## Results

### CRISPR/Cas9-editing and induction of *KMT2A*-rearranged chromosomal translocations appears to be proliferation dependent

**Fig. 1:**
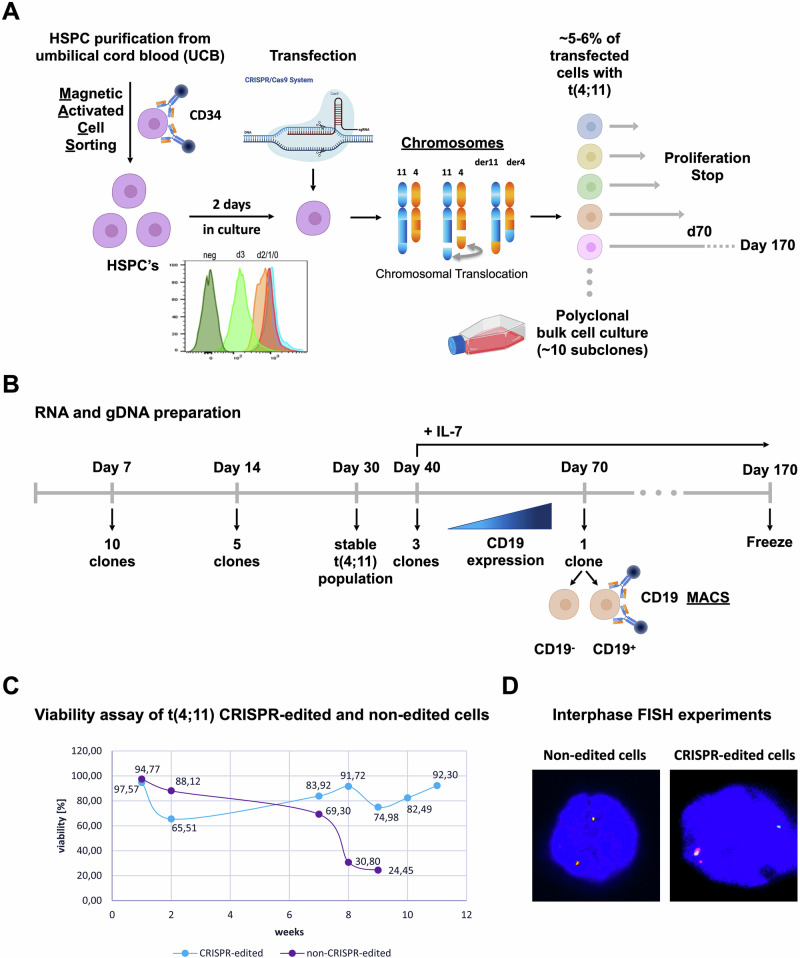
Experimental setup for the induction of the t(4;11) chromosomal translocation in UCB HSPCs. **A** CD34^+^ HSPC purification from umbilical cord blood (UCB), transfection and CRISPR/Cas9-editing of *KMT2A/MLL* intron 9 and *AFF1/AF4* intron 3 on day 2 of culture. CellTrace^TM^ Proliferation assay of CD34^+^ HSPCs MACS-separated from umbilical cord blood on day 0 (blue peak), day 1 (red peak), day 2 (orange peak) and day 3 (light green peak) of culture. Negative control (dark green peak) comprised unstained cells. Flow cytometry analysis of the staining intensity. Clonality analysis of the genomic DNA (gDNA) breakpoint region of t(4;11) translocated cells. **B** Timeline of gDNA and RNA preparation of t(4;11) translocated cells on day 7, 14, 30, 40, and 70 post-transfection. IL-7 stimulation was performed upon lineage specific differentiation into the lymphoid CD19^+^ B cell direction. CD19^+^ cells were separated by magnetic activated cell sorting (MACS). **C** Viability assay of t(4;11) CRISPR/Cas9-edited and non-edited UCB HSPCs evaluated by 7AAD flow cytometry. **D** Interphase fluorescence in situ hybridization (FISH) of non-edited HSPCs and t(4;11) CRISPR/Cas9-edited HSPCs 2 days post-transfection. A yellow signal represents an intact *KMT2A* gene, a green and red signal represents a chromosomal translocation of the *KMT2A* gene. The figure was generated with Biorender, FlowJo analysis, and ImageJ.

Here, we applied the recently established CRISPR/Cas9 system [31]. Freshly MACS-isolated CD34^+^ HSPCs were kept in medium for at least 2 days before CRISPR/Cas9-editing, because the induction of chromosomal translocations appeared to be proliferation or cell cycle dependent (see small inset in Fig. 1A). At day 0 and day 1, CRISPR/Csa9-editing was not successful and no *KMT2A*-rearrangement was detectable (data not shown). However, nucleofection on day 2 of culture, resulted in a high percentage of nearly 5% of t(4;11) translocations. Moreover, we analyzed the composition of HSPC-derived sub-populations on day 0, 1 and 2 by flow cytometry [34–36]. As summarized in Supplemental Fig. 1, Lin^-^ CD34^+^ CD38^-^ showed three different sub-populations (HSCs: CD45RA^-^ CD90^+^; MPPs: CD45RA^-^ CD90^-^; LMPPs: CD45RA^+^ CD90^-^). On day 0, the vast majority of cells were either HSCs or MPPs. However, on culture cells became CD34^+^ CD38^+^ and differentiated into the MPP, LMPP, GMP, CMP, MEP, and CLP progenitor compartments although no cell division occurred until day 2. This phenotypic change was accompanied by the increase in cell size (FSC) and granularity (SSC). These results underline that the induction of the chromosomal translocation may be proliferation dependent and that not only HSCs are nucleofected but also many different progenitor populations.

### Establishment of long-term cultures of ex vivo manipulated umbilical cord blood HSPCs

MACS-purified HSPCs were cultured for 2 days in StemSpan SFEMII medium, supplemented with Pen-Strep, donor plasma, SCF, FLT3L and TPO. These proliferating cells were subsequently nucleofected with *KMT2A-* and *AFF1-specific* sgRNA/Cas9-Ribonuleoproteins (RNPs), as recently described [31] (see Fig. 1A). The number of t(4;11)-rearranged cells was quantified on day 3 by genomic RT-PCR experiments to be in the range of 5-6% (data shown in Benz et al. 2025) [31]. The genetically modified cells were kept in co-culture with the non-genetically modified cells and molecular testing revealed a nearly 100% purity of t(4;11)-translocated cells around day 30, while non-genetically modified HSPCs were outgrown and absent at this timepoint (see Fig. 1A and B). Interestingly, another culture of non-genetically modified cells displayed viability until week 8, and then lost viability (see Fig. 1C), while the t(4;11)-positive population stably expanded from 2 weeks onwards. Interphase FISH experiments were also performed at day 4 of culture, where single cells were found to contain the specific t(4;11) rearrangements (example is shown in Fig. 1D).

**Fig. 2:**
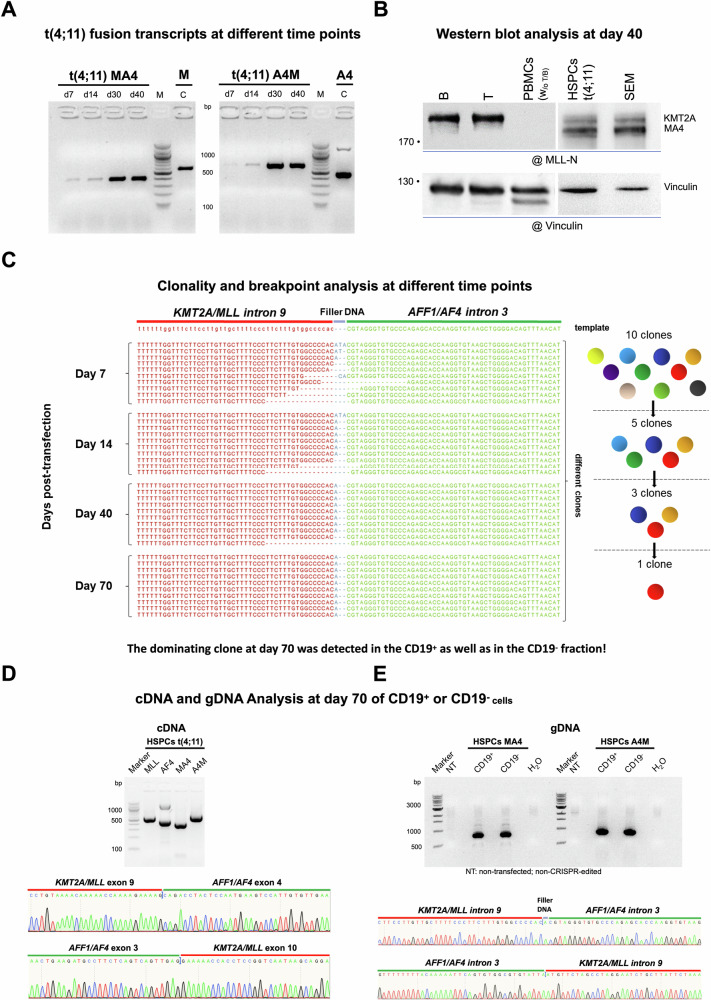
The detection of t(4;11) fusion transcripts, the fusion protein and gDNA clonality analysis. **A** The detection of the *KMT2A/MLL::AFF1/AF4* (MA4) and *AFF1/AF4::KMT2A/MLL* (A4M) fusion transcripts and endogenous *KMT2A/MLL* (M) and *AFF1/AF4* (A4) transcripts on day 7, 14, 30 and 40 of culture. **B** Western Blot analysis at day 40 post-transfection for the detection of the KMT2A/MLL wildtype protein and KMT2A/MLL::AFF1/AF4 fusion protein in B cells, T cells, PBMCs (without (w/o) B and T cells), t(4;11) HSPCs and SEM cells (as control cell line). Vinculin (124 kDa) served as loading control. KMT2A/MLL wildtype: 300 kDa; KMT2A/MLL::AFF1/AF4 fusion protein: ~245 kDa. **C** gDNA clonality analysis of culture days 7, 14, 40, and 70. Out of 10 clones at day 7 only one clone became dominant over the culture period. **D** cDNA analysis of the wildtype and fusion transcripts of day 70 CD19^+/-^ t(4;11) cells. Sanger sequencing revealed fusion transcript sequences. **E** gDNA analysis of day 70 CD19^+^ and CD19^-^ t(4;11) cells and sanger sequencing of the purified PCR-bands. The figure was generated with ImageJ, CLC Sequence Viewer, and SnapGene.

Semi-quantitative RT-PCR analyses (only 25 cycles) were also performed with a small RNA sample obtained at days 7, 14, 30 and 40 to demonstrate that the amount of fusion transcripts increased over time and stabilized by day 30 (see Fig. 2A). In addition, Western blot analysis revealed the presence of the KMT2A::AFF1 fusion protein at day 40, in comparable amounts as expressed in the t(4;11) cell line SEM. Wildtype KMT2A was also observed in purified B- or T-cells, while remaining PBMC’s depleted of B- and T-cells remained negative due to the fact that many cells in the PBMC fraction do not express the KMT2A protein (see Fig. 2B).

### Clonality analysis revealed subclone evolution over time

Genomic PCR experiments were also performed at different timepoints (days 7, 14, 40 and 70). Briefly, isolated genomic DNA was used for genomic PCR experiments. Subsequently, these PCR fragments were subcloned in the Topo pCR2.1 cloning vector and subjected to Sanger sequencing to reveal the number of different clones that were created during the Cas9 cleavage and DNA repair process. A total of 20 clones were analyzed from each of these 4 timepoints. As shown in Fig. 2C, oligoclonality of the induced t(4;11) translocations could be shown for day 7, where 10 different subclones were identified. These subclones disappeared over time (5 subclones remaining on day 14, 3 subclones on day 40 and 1 subclone on day 70). Thus, the final clone carried an additional A-nucleotide (filler DNA deriving from the NHEJ mediated DNA repair process) between the sequences derived from *KMT2A* intron 9 and *AFF1* intron 3, respectively (see Fig. 2C and E).

**Fig. 3:**
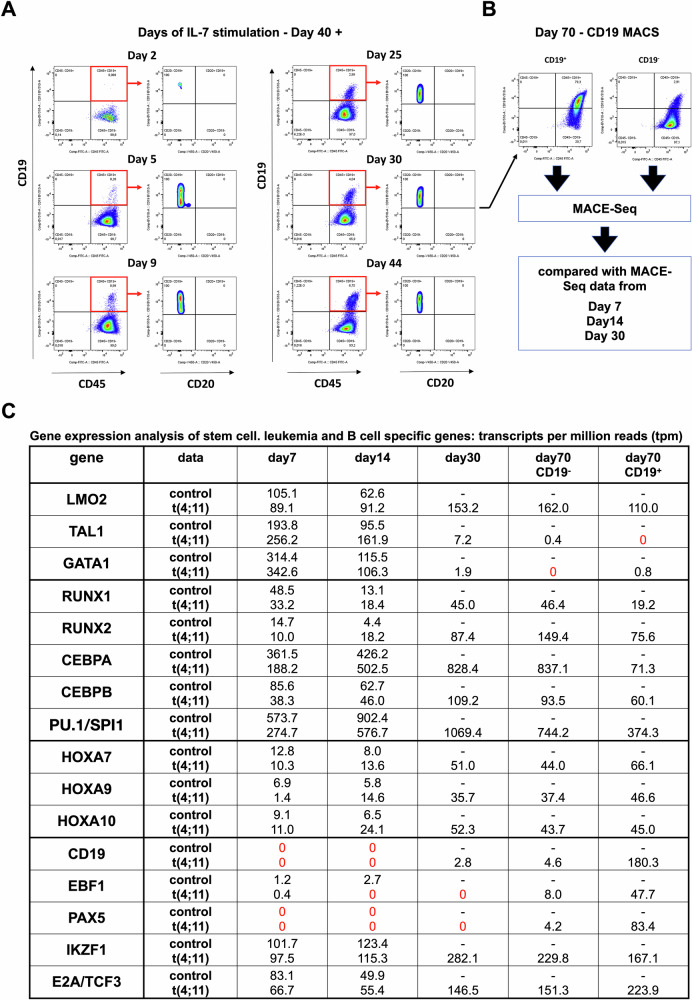
Upregulation of the CD19 receptor and gene expression analysis of stem cell like, B cell and leukemia specific genes. **A** Flow cytometry analysis of CD45^+^ CD19^+^ and CD19^+^ CD20^-^ cells due to IL-7 stimulation over 44 days. Gating strategy: FSC/SSC population, single cells, viable 7AAD^-^ cells, x-axis: CD45 y-axis: CD19, from the CD45^+^ CD19^+^ cell population gated to x-axis: CD19 y-axis: CD20. **B** MACS separation of CD19^+^ and CD19^-^ t(4;11) cells after 30 days of IL-7 stimulation (day 70 in culture). The CD19^+^ and CD19^-^ fractions were used for MACE-Seq analysis and compared with day 7 and day 14 of the non-CRISPR/Cas9-edited HSPCs from the same donor than day 7, 14 and 30 of the t(4;11) CRISPR/Cas9-edited HSPCs. **C** Analysis of stem cell like genes, B cell and leukemia specific genes with the transcript per million (tpm) mean values of day 7 and day 14 non-CRISPR/Cas9-edited (control) and day 7, 14, 30, 70 CD19^+^ and 70 CD19^-^ t(4;11). The figure was generated with FlowJo.

From day 40 onwards, the oligoclonal bulk culture was treated with additional IL-7 in order to study the potential plasticity of these manipulated cells (see Fig. 1B and Fig. 3A). We assumed that the almost pure cell population might have the capacity to respond to external signals with appropriate differentiation into the lymphoid, or B-cell lineage. At day 70, the bulk culture was MACS sorted for CD19 expressing cells. Cells could be separated into approximately 6% CD19^+^ and 94% CD19^-^ cells in the bulk cell culture (see Fig. 3A and B). Thus, only a small portion of the bulk culture was able to respond to this exogenous IL-7 signal and to differentiate into proB-like cells, while 94% of all cells at day 70 remained undifferentiated, with activated genes known to be expressed in myeloid cells (*FLT3, IDH1/2, NPM1, CEBPA, TP53, RUNX1, ASXL1, DNMT3A, SRSF2, U2AF1, SOX4*, *CD86* and *JAK2*) but also with genes known to be expressed in lymphatic cells (*CD9*, *CD72*, *CD79A/B*, *CD109*, *IGHM, IGHL, IL12R, TCF3, IKZF1, NOTCH1, VAV2* and *CCL5*). Finally, cells were expanded until day 170 and then cryopreserved in liquid nitrogen.

Cells at day 70 were subjected again to RT-PCR analysis to demonstrate that these cells express *KMT2A* (*MLL*), *AFF1* (*AF4*) as well as *KMT2A*::*AFF1* (MA4) and *AFF1*::*KMT2A* (A4M) transcripts (see Fig. 2D). We also tested for the specific rearrangements of *KMT2A* intron 9 and *AFF1* intron 3 by genomic PCR, to demonstrate that the CD19^+^ and CD19^-^ cell populations were genetically identical. Subsequent sequencing analysis revealed the correct splicing of the two fusion gene mRNAs, but also the presence of the identical fusion alleles (*KMT2A::AFF1* and *AFF1::KMT2A*) in both cell populations (see Fig. 2E).

### Flow cytometry analyses revealed an IL-7-mediated onset of CD19 surface expression in the CRISPR/Cas9-generated t(4,11) cells

From different time points of the bulk culture, we used flow cytometry analysis to monitor the induction of CD19, while CD20 was completely absent in the CD45^+^ cell population of the CRISPR/Cas9-edited t(4;11) cells. CD19 positivity was used as a surrogate marker to trace t(4;11) translocated cells that developed into the B-cell lineage. As shown in Fig. 3A, very few cells were visible on day 2 of IL-7 stimulation, while from day 5 onwards, a robust and growing CD19^+^ cell population became visible (Days of IL-7 stimulation: day 2: 0.07%; day 5: 0.26%; day 9: 0.99%; day 25: 2.99%; day 30: 4.04%; day 44: 6.75%). On day 30 of IL-7 stimulation (day 70 of cell culture), the cells were MACS sorted into CD19^+^ and CD19^-^ fractions (CD19^+^ cells ~80%; CD19^-^ cells ~3%) (see Fig. 3B). It is interesting to note that gene expression profiling (GEP) on days 7, 14, 30 and 70 of the CD19^-^ population revealed virtually no *PAX5* expression, however, *PAX5* became significantly expressed at day 70 in the CD19^+^ population of cells (83.4 tpm reads in the CD19^+^ fraction vs. 4.1 reads in CD19^-^ fraction) (see Fig. 3C). However, CD19 gene expression was already observed at day 30 (2.8 tpm reads) and became strongly enhanced at day 70 in the CD19^+^ cells (180.3 tpm reads in the CD19^+^ fraction vs. 4.6 reads in the CD19^-^ fraction). It is probable that CD19 is not only a direct target gene of PAX5, but also of a transcription factor that became upregulated upon addition of the IL-7 cytokine. Apart from *TAL1* and *GATA1*, all expressed genes showed a decline over time in the non-edited cells, but an increase in expression of the same genes in the t(4;11) cell population.

### Flow cytometry-characterization of the CRISPR/Cas9-generated t(4;11) and non-genetically modified cells after 40 days of IL-7 treatment

**Fig. 4:**
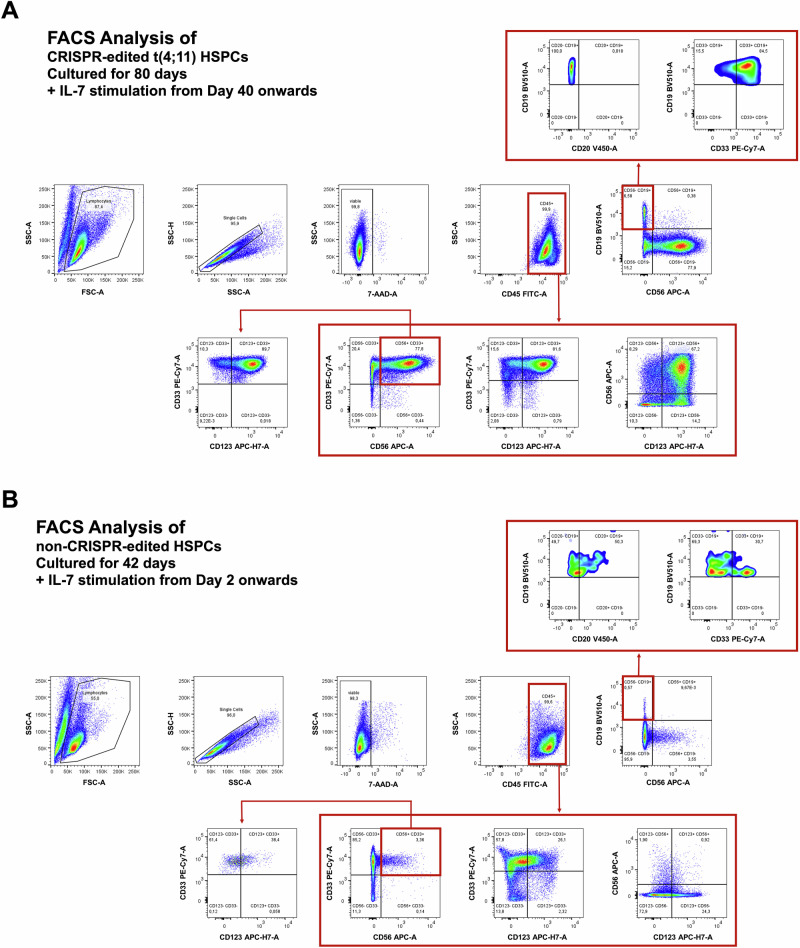
Phenotype analysis of t(4;11) HSPCs and non-CRISPR/Cas9-edited HSPCs. **A** t(4;11) HSPCs were analyzed on day 80 of culture and after 40 days of IL-7 stimulation. The gating comprised single cells, viable 7AAD^-^ cells, and C45^+^ cells. **B** Non-CRISPR/Cas9-edited HSPCs were analyzed on day 42 of culture and after 40 days of IL-7 stimulation. The gating comprised single cells, viable 7AAD^-^ cells, and C45^+^ cells. The Figure was generated with FlowJo.

We also compared the Cas9-generated t(4;11) cells with wildtype HPSCs that were also treated with IL-7 for 40 days. As summarized in Fig. 4A, viable single cells were stained for CD45 and sub-gated into the CD56^-^/CD19^+^ fraction (6.5%) to demonstrate the absence of CD20 expression, but the co-expression of CD33 on these CD19^+^ cells, similar to the mixed-lineage-leukemia pro-B phenotype well-known from t(4;11) patients. In the CD56^+^/CD19^-^ cell population (78.2%) we observed co-expression of CD123 and CD33 in the majority of cells (> 70% myeloid lineage phenotype). A similar analysis with non-genetically modified HSPCs that were stimulated also for 40 days with IL-7 in cell culture displayed only a small fraction (0.57%) of CD56^-^/CD19^+^ cells which displayed the co-expression of CD20 and CD33 (see Fig. 4B). The majority of cells were CD56^-^/CD19^-^ (96%) and displayed co-expression of CD33 whereas 26% were CD123 positive as well. CD56^+^ cells were rare (3.5%) and were mostly CD123 negative, but CD33 positive. In conclusion, the presence of the Cas9-mediated t(4;11) translocation caused upregulation of CD19, typically found in proB ALL cells, co-expression of CD33, as found in many patients, but the absence of CD20. However, we also found a fraction of myeloid cells expressing CD123, CD56 and CD33. This fraction resembles the population of myeloid lineage cells after treatment of cultures with IL-7 over 40 days. In non-t(4;11) cells we did have a major fraction of cells that was positive for CD123 and CD33, but negative for CD19 and CD56.

### Gene expression analysis of CD19^+^ and CD19^-^ cells after 70 days of in vitro cell culture

**Fig. 5:**
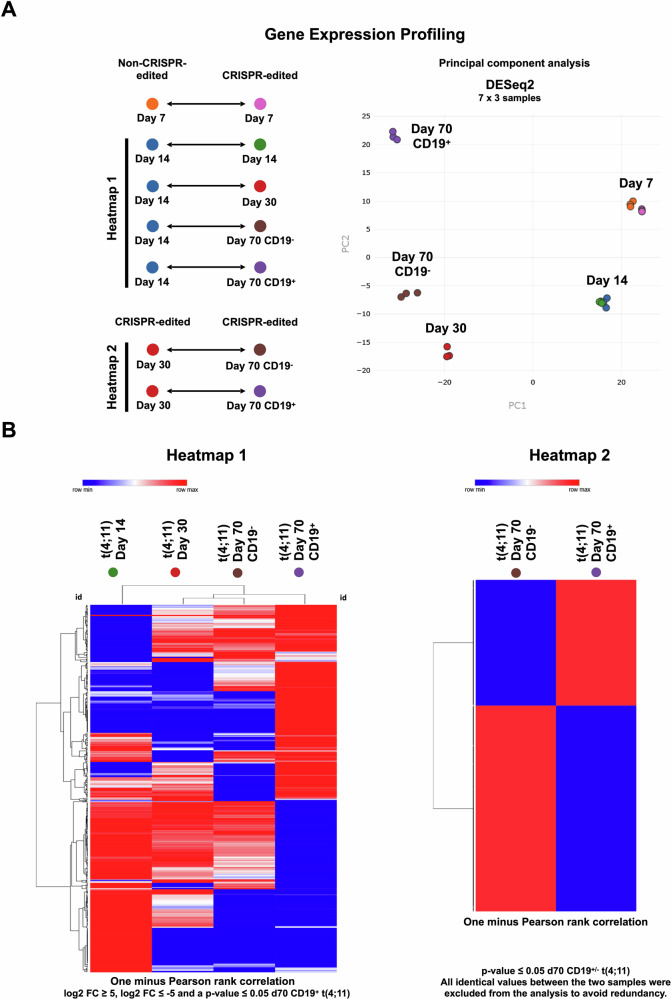
Gene expression profiling (1) of t(4;11) CRISPR/Cas9-edited UCB HSPCs over time. **A** General overview of the gene expression data comparison of non-CRISPR/Cas9-edited cells and t(4;11) CRISPR/Cas9-edited cells on the days 7, 14, 30, 70 CD19^+^ and 70 CD19^-^ (heatmap 1). Comparison of gene expression data from day 30 t(4;11) cells and day 70 CD19^+^ and CD19^-^ cells was performed for heatmap 2. Principle component analysis (PCA) showed the clustering of the triplicates of all 21 samples. **B** Heatmap 1 was generated based on log2FC values ≥ 5 and ≤ -5 of the comparison of day 14 vs. day 14 t(4;11), day 30 t(4;11), day 70 CD19^-^ t(4;11) and day 70 CD19^+^ t(4;11) and significant values with a *p* value ≤ 0.05 based on day 70 CD19^+^ t(4;11) sample for a better lineage comparison. Heatmap 2 was generated with log2FC values based on the comparison of day 30 t(4;11) vs. day 70 CD19^+^ t(4;11) and day 70 CD19^-^ t(4;11). All identical values were excluded to avoid redundancy. The hierarchical clustering for both heatmaps, heatmap 1 and 2, was performed with the one minus Pearson rank correlation. The figure was generated with the web tool Morpheus heatmap.

As outlined in the material and methods section, we analyzed the differential gene expression on several days of culture. We did not observe any changes in gene expression on day 7 and day 14 (data not shown). The first differences were clearly visible on day 30 and on day 70 when comparing the CD19^+^ and CD19^-^ cell populations. We performed different comparisons and PCA analyses (Fig. 5A) and summarized our findings in 2 heatmaps shown in Fig. 5B. Heatmap 1 clearly shows the development of specific gene signatures, and the largest changes in the CD19^+^ cell population. Heatmap 2 shows the signature of the CD19^+^ and CD19^-^ cell populations compared to day 30. A comparison of important stem cell genes and therie changed expression is summarized in Table 2.

**Table 2:** Mean transcript per million (tpm) reads of specific stem cell genes.

gene name	HSC d7	HSC d14	HSC d7 t(4;11)	HSC d14 t(4;11)	HSC d30 t(4;11)	HSC d70 CD19^-^ t(4;11)	HSC d70 CD19^+^ t(4;11)
**ACSM3**	20,21	4,03	3,16	10,64	2,42	18,71	0,00
**ALDH1A1**	3,60	6,06	8,51	9,68	0,00	0,00	0,00
**ANKRD28**	13,81	4,94	4,98	7,95	4,53	6,30	5,50
**ARMCX1**	3,60	2,67	2,87	2,43	0,00	0,00	0,00
**AVP**	1,52	0,00	0,00	0,00	0,00	0,00	0,00
**BEX1**	2,50	23,26	0,00	41,11	0,00	0,00	0,00
**BEX2**	8,72	0,00	5,71	0,00	0,00	0,00	0,00
**BST2**	299,66	583,65	242,49	459,25	728,59	846,36	716,42
**CD164**	67,27	79,92	72,86	95,84	78,01	62,46	87,39
**CD34**	15,07	3,08	3,92	13,78	17,80	0,00	0,00
**CFH**	12,03	0,00	4,61	0,00	0,00	0,00	1,63
**CLEC9A**	2,11	0,00	0,00	0,00	3,74	0,00	31,93
**CLU**	3513,71	1482,26	5447,49	858,11	25,16	10,27	2,51
**CRHBP**	1,05	0,00	0,00	0,00	0,00	0,00	0,00
**DLK1**	37,88	2,32	29,94	0,00	0,00	0,00	0,00
**EGFL7**	109,43	93,05	112,14	83,36	18,66	10,31	82,00
**HOPX**	1,20	0,00	1,79	2,28	2,83	0,00	0,00
**HSD17B14**	0,00	19,86	2,15	0,00	0,00	0,00	0,00
**IDS**	0,00	0,00	0,00	1,21	2,84	5,59	0,00
**IL18**	43,75	12,12	17,84	17,70	20,48	24,66	24,23
**KIT**	28,38	35,16	18,75	52,81	8,22	0	0
**KRT18**	5,37	0,00	3,57	0,00	0,00	0,00	0,00
**KRT8**	14,09	0,00	11,79	0,00	0,00	0,00	0,00
**LDOC1**	3,27	0,00	2,83	2,69	0,00	0,00	0,00
**LMO2**	105,06	62,57	89,12	91,18	153,15	169,21	109,97
**MDK**	13,93	0,00	0,00	0,00	3,27	9,73	0,00
**MECOM**	3,29	0,00	4,29	0,00	2,51	0,00	0,00
**MEG3**	21,63	0,00	20,13	4,68	0,00	0,00	0,00
**MLLT3**	26,62	4,94	30,00	17,91	4,93	4,19	29,09
**MMRN1**	148,11	13,92	122,65	52,49	0,00	0,00	0,00
**MSRB3**	9,83	25,27	12,04	22,42	0,00	0,00	0,00
**MYCT1**	45,77	0,00	38,71	26,03	0,00	0,00	0,00
**NPR3**	10,55	0,00	9,64	9,52	5,46	0,00	25,55
**NRIP1**	24,73	8,22	25,15	13,21	41,85	27,57	21,93
**PCDH9**	2,73	0,00	0,00	0,00	0,00	0,00	11,33
**PRDX1**	73,76	181,66	141,21	316,89	386,63	174,73	697,58
**PRKG2**	0,00	0,00	2,47	0,00	0,00	0,00	0,00
**PROM1**	7,41	0,00	2,16	5,52	25,78	5,95	43,76
**RBPMS**	7,16	0,00	0,00	5,21	2,97	0,00	0,00
**SOCS2**	39,31	11,02	38,08	37,13	26,18	119,01	985,02
**SPI1**	573,65	902,44	274,69	576,69	1069,44	744,20	374,29
**SPINK2**	11,05	7,03	4,22	16,93	11,15	1,37	34,79
**SYPL1**	38,61	32,94	42,30	53,71	33,93	22,33	42,60
**TAL1**	193,78	95,49	256,19	161,93	7,19	0,00	0,00
**TSC22D1**	20,41	14,40	18,43	19,90	8,62	3,10	50,25
**UCHL1**	0,00	0,00	0,00	3,25	0,00	0,00	0,00

**Fig. 6:**
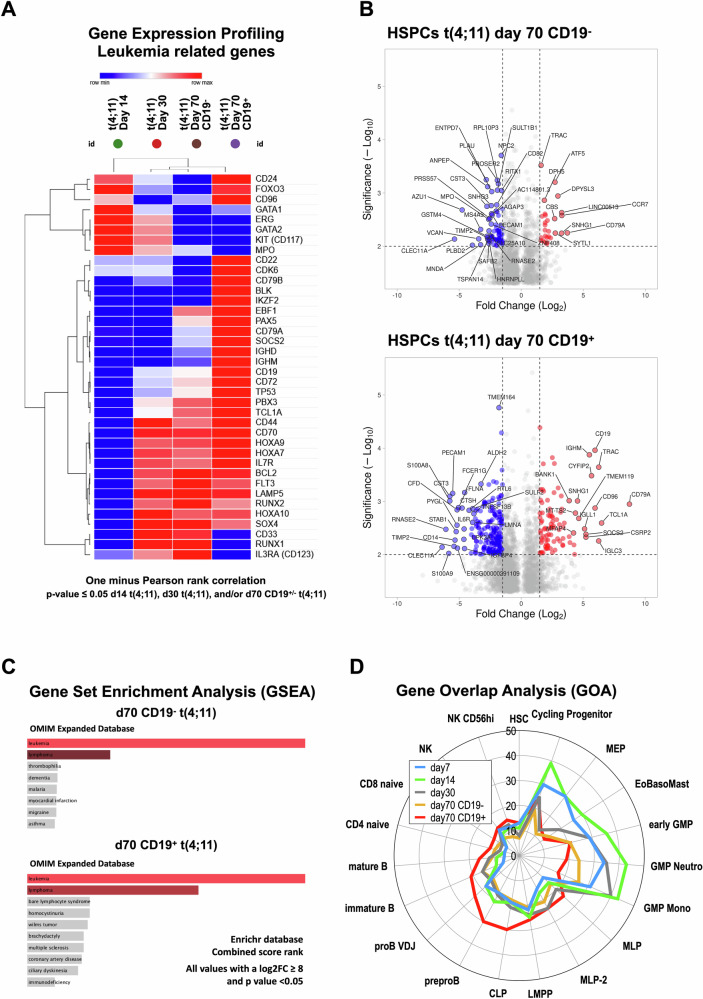
Gene expression profiling (2) of t(4;11) CRISPR/Cas9-edited UCB HSPCs over time. **A** Gene expression analysis of leukemia related genes was performed with Log2FC values of day 14 vs. day 14 t(4;11), day 30 t(4;11), day 70 CD19^-^ t(4;11) and day 70 CD19^+^ t(4;11). Based on these log2FC values a heatmap was generated with *p* values ≤ 0.05 either from day 14 t(4;11), day 30 t(4;11), day 70 CD19^-^ t(4;11) and/or day 70 CD19^+^ t(4;11). The hierarchical clustering for the heatmap was performed with the one minus Pearson rank correlation. The figure was generated with the web tool Morpheus heatmap. **B** Volcano plots of HSPCs t(4;11) day 70 CD19^-^ and HSPCs t(4;11) day 70 CD19^+^ were generated with the log2FC values and the corresponding minus log10 *p* values. log2FC values were calculated based on the comparison of day 30 t(4;11) vs. day 70 CD19^-^ t(4;11) and day 70 CD19^+^ t(4;11). Only values were used that had a tpm mean ≥ 2 and significant *p* values ≤ 0.05. The volcano plots were generated with VolcanoNoseR. **C** Gene set enrichment analysis (GSEA) was performed for HSPCs t(4;11) day 70 CD19^-^ and HSPCs t(4;11) day 70 CD19^+^ in comparison to day 14 of non-CRISPR/Cas9 edited cells. Only significant *p* values ≤ 0.05 and high log2FC values of ≥ 8 were used for the analysis. Data of the combined score rank of the OMIM Expanded Database showed the associated diseases phenotype. The figure was generated with Enrichr. **D** Radar chart evaluation for gene overlap analysis (GOA) of top marker genes for specific hematopoietic cell states. Top marker genes (max 50) derived from Zeng et al. [37] and were scored positive when overlapping genes from our signatures had at least 20 tpm and a *p*-value of < 0,05.

In Fig. 6A, we displayed a heatmap of selected leukemia-specific genes, the corresponding volcano plots (see Fig. 6B) as well as the gene enrichment analyses (see Fig. 6C) and a gene overlap analysis (GOA) over time based on top marker genes for each hematopoietic cell type (see Fig. 6D) [37]. The CD19^-^ and CD19^+^ cell populations commonly express a *HOXA* signature (*HOXA7*, *HOXA9*, *HOXA10*), *MYB*, *PBX3*, *FLT3*, *CD117/KIT*, *BCL2*, *RUNX2*, *SOX4* and *CD44*. Weaker signals were found for *MEIS1*, *CD133/PROM1* and *CSPG4/NG2*. The CD19^-^ population expressed *ATF5*, CCR7 and *CD79A*, while the CD19^+^ population highly expressed *CD79A/B*, *CD96*, *CD19*, *IGHM*, *TCL1A*, *IGLC3*, *IGLL1*, *SOCS2*, *TMEM119*, *CYFIP2*, *IL7R*, *CD72*, *PAX5* and *CDK6*, displaying a clear bias into the B-cell lineage. GSEA analysis revealed that the obtained signatures at day 70 (CD19^-^ and CD19^+^) are associated with leukemia and lymphoma signatures. We also compared our signatures from day 7, day 14, day 30 and both signatures from day 70 (CD19^-^ and CD19^+^) with the recently published atlas of subpopulations in the human hematopoietic compartment [37]. The radar chart analysis performed with the “top 50” genes of all these different compartments revealed that the CD19^-^ population on day 70 is highly similar to the early GMP and the development into neutrophils and monocytes, while the CD19^+^ population shows a clear similarity to LMPP, CLP and very early B cell committed cells. This clearly confirms our assumption that cells at day 70 are either committed into the myeloid lineage or were able to respond to the external IL-7 signals to commit into a lymphoid or B-cell lineage. This kind of cellular plasticity at day 40 of these ex vivo cultured cells was unexpected.

**Fig. 7:**
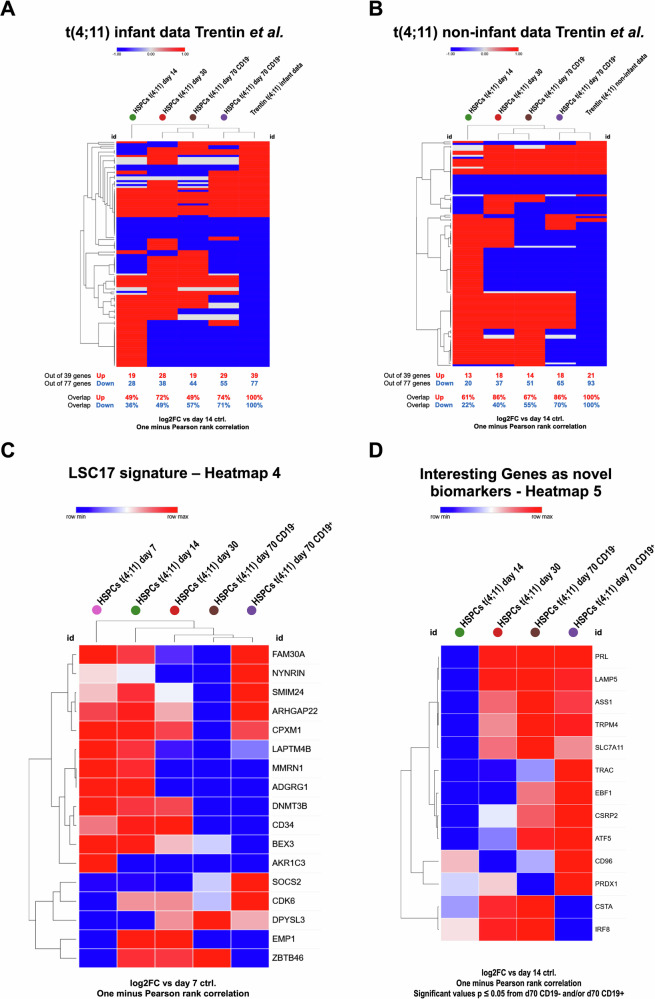
Gene expression profiling analysis. **A** Comparison of t(4;11) infant leukemia patient (age < 1 year) data derived from Trentin et al. and t(4;11) CRISPR/Cas9-edited UCB HSPC data of day 14, day 30, day 70 CD19^-^ and day 70 CD19^+^. A certain set of up and down regulated genes from Trentin et al. was used for the comparison. Log2FC data were used for the comparison. Definition of evaluation: upregulated genes = 1, unchanged genes = 0, downregulated genes = - 1 [20]. The hierarchical clustering for the heatmap was performed with the one minus Pearson rank correlation. The figure was generated with the web tool Morpheus heatmap. **B** Comparison of t(4;11) non-infant leukemia patient (age > 1 year) data derived from Trentin et al. and t(4;11) CRISPR/Cas9-edited UCB HSPC data of day 14, day 30, day 70 CD19^-^ and day 70 CD19^+^. A certain set of up and down regulated genes from Trentin et al. was used for the comparison. Log2FC data were used for the comparison. Definition of evaluation: upregulated genes = 1, unchanged genes = 0, downregulated genes = - 1 [20]. The hierarchical clustering for the heatmap was performed with the one minus Pearson rank correlation. The figure was generated with the web tool Morpheus heatmap. **C** The leukemia stem cell signature 17 (LSC17) was applied to the t(4;11) CRISPR/Cas9-edited UCB HSPCs over time. Gene expression analysis of leukemia related genes was performed with Log2FC values of day 7 vs. day 7 t(4;11), day 14 t(4;11), day 30 t(4;11), day 70 CD19^-^ t(4;11) and day 70 CD19^+^ t(4;11). The hierarchical clustering for the heatmap was performed with the one minus Pearson rank correlation. **D** Interesting target genes as biomarker for t(4;11) transformed cells. Gene expression analysis of interesting target genes was performed with Log2FC values of day 7 vs. day 7 t(4;11), day 14 t(4;11), day 30 t(4;11), day 70 CD19^-^ t(4;11) and day 70 CD19^+^ t(4;11). The hierarchical clustering for the heatmap was performed with the one minus Pearson rank correlation. Based on these log2FC values a heatmap was generated with *p*values ≤ 0.05 for day 70 CD19^-^ and/or CD19^+^. The figures were generated with the web tool Morpheus heatmap.

Further, we compared our t(4;11) CRISPR-edited UCB HSPC data to data derived from t(4;11) infant leukemia and t(4;11) non-infant leukemia patients (see Fig. 7 A and B) [20]. Infants were defined with an age < 1 year. Non-infants were defined with an age > 1 year. It is clearly demonstrated that the day 70 CD19^+^ fraction has a great overlap with specifically up and down regulated genes in t(4;11) infant leukemia patients (74% overlap of upregulated genes, 71% overlap of downregulated genes), as well as in t(4;11) non-infant leukemia patients (86% overlap of upregulated genes, 70% overlap of downregulated genes). Upon closer examination, a great overlap of the t(4;11) patient data with the t(4;11) day 30 sample can be already detected at an early cell culture stage without B cell lineage commitment. This nicely shows the cancer evolution during the different time points and reveals the pre-leukemic state of the in vitro CRSIPR/Cas9-edited cells compared to the t(4;11) infant and non-infant patient data. Thus, this CD19^+^ subpopulation most likely mimicked already the situation in t(4;11) leukemia patients. We also compared our signatures with the LSC17 signature (as summarized in Fig. 7C).

All data for creating these heatmaps, volcano plots and GSEA analysis are available as Supplementary Excel files 2–11.

## Discussion

Here, we successfully demonstrate that the generation of specific *KMT2A*-rearrangements via nucleofection of pre-loaded sgRNA/Cas9 ribonucleoprotein (RNP) complexes is an easy and feasible way to create model systems for *KMT2A*-rearrangements. We created an oligoclonal and bona fide t(4;11)(q21;23) chromosomal translocation in hematopoietic stem and precursor cells (HSPCs) deriving from umbilical cord blood (UCB). This CRISPR/Cas9 model represents a reliable method to compare single hit t(4;11) transformed cells to healthy non-transformed cells of the same cord blood donor. As present in the patients, our model is proved for the expression of both fusion transcripts, *KMT2A/MLL::AFF1/AF4* and *AFF1/AF4::KMT2A/MLL*, presuming a leukemic outgrowth of the cells [16]. In order to study the resulting molecular effects, we used differential gene and cell surface marker expression to gain insights into the very early steps of cell fate decisions. Based on the investigated markers, we traced the conversion of healthy HSPCs into pre-leukemic cells (CD19^-^), and cells that most likely mimic a leukemic phenotype (CD19^+^). These cells could be expanded until day 170 ex vivo before their cryo-preservation. Unmodified HSPC cells were outcompeted between day 30 and 56 of culture in independent experiments (*n* = 3). Some stem cell markers, such as *TAL1* and *GATA2*, vanished rapidly in the newly generated t(4;11) cells, while other markers such as *LMO2*, remained [38–40]. Surface markers such as FLT3, CD133, NG2 and CD72 were stably expressed on these t(4;11) cells. Interestingly, we observed that CD56 was upregulated in t(4;11) CRISPR-edited cells stimulated with IL-7. Normally, CD56 is a well-known NK cell marker, but it has been demonstrated that CD56 is co-expressed in aggressive forms of leukemia as well, especially in AML [41–43]. In 20% of AML patients, CD56 was observed on leukemic blasts and correlated with chemotherapy resistance [44]. The CD19^+^ t(4;11) cell population displayed a clear leukemic signature, indicating that they rapidly evolved from day 30 of the bulk cell culture by an exogenous IL-7 stimulation. An established signature of selected leukemia stem cell genes (*n* = 17) (LSC17 signature) was used to analyze leukemia stem cell features in those t(4;11) CRISPR-edited HSPCs over time [45, 46]. Many leukemia stem cell like genes of the LSC17 signature diminished after day 14 such as *LAPTM4B*, *MMRN1, ADGRG1* or *AKR1C3*, but some were upregulated again by day 30, day 70 CD19^-^ and/or day 70 CD19^+^. LSC genes such as *FAM30A, NYNRIN, ARHGAP22, CPXM1, SOCS2* or *CDK6* regained function and were highly expressed within the CD19^+^ population on day 70, whereas the majority of LSC genes was downregulated in the CD19^-^ population on day 70 (see Fig. 7C) These transcription data demonstrate that leukemia stem cell like genes are not only present within the first 7 to 14 days of culture. Depending on the fraction CD19^+^ or CD19^-^, different stem cell related genes were expressed again on day 70, unveiling the cancerous potential of these pre-leukemic CRISPR-edited cells (see Fig. 7). The upregulation of hematopoietic stem cell genes may also be an indication that the cell of origin is a leukemic stem cell (LSC). The strongest clone detectable from day 30 onwards may have been a t(4;11) transformed LSC outcompeting the t(4;11) transformed progenitor cells (MPPs, LMPPs, GMPs, CMPs, MEPs, CLPs).

This indicates that these ex vivo created t(4;11) cells are enriched for stem cell genes, even when cultured ex vivo. This is also reflected by the GSEA analysis shown in Fig. 6C, where the signatures clearly overlapped with leukemia or lymphoma signatures [47–49]. IL-7 as a sole external and additional signal was able to drive a portion of these cells into the lymphoid lineage or proB cell state (CD19^+^), while the remaining cells (CD19^-^) remained in a state that resembles the myeloid lineage (see Fig. 6D).

The bulk population at day 30 and the CD19^-^ population at day 70 may still represent a pre-leukemic population of cells in which cell fate decisions have not been taken. Since the majority of the ex vivo cultured t(4;11) cells are CD19^-^, it may indicate that appropriate signals necessary for the development into leukemic cells are missing. These signals usually derive usually from the bone marrow niche, however, are missing in our experimental setting. However, a small portion of cells was able to develop into a CD19^+^ cell population, simply by the presence of the IL-7 cytokine. This subpopulation most likely reflected the gene expression of t(4;11) patients (see Fig. 7A and B).

Further, we discovered a set of significantly upregulated genes that were exclusively expressed in our t(4;11) CRISPR-edited HSPCs and might display possible new and interesting targets (see Fig. 7D). This gene set comprises *PRL, LAMP5, ASS1, TRPM4, SLC7A11, TRAC, EBF1, CSRP2, ATF5, CD96, PRDX1, CSTA* and *IRF8*.

In conclusion, Cas9-mediated chromosomal translocations of the *KMT2A* gene in UBC HSPCs represent novel tool for studying pre-leukemia development and probably also leukemia onset ex vivo. Comparing several of such systems carrying different *KMT2A* rearrangements will help us to understand the oncogenic mechanisms in greater detail. These early events were previously impossible to investigate, because genetically modified cells (retroviral/lentiviral) are usually directly transplanted into mice in order to observe in vivo leukemia development in a reasonable timeframe.

Several labs have recently set-up such CRISPR/Cas9 experiments for different *KMT2A* translocation and have used this novel technology to create experimental model systems in order to study particular types of leukemia diseases [28, 29, 50, 51]. All these data clearly demonstrate important insights in leukemia disease development. Comparing different *KMT2A* translocations in the future will help to unravel the basic mechanisms of cell fate reprogramming of this particular group of high-risk acute leukemia patients.

## Supplementary information

Supplementary Figure S1

Supplementary Excel file S2

Supplementary Excel file S3

Supplementary Excel file S4

Supplementary Excel file S5

Supplementary Excel file S6

Supplementary Excel file 7A

Supplementary Excel file S7B

Supplementary Excel file S8

Supplementary Excel file S9

Supplementary Excel file S10

Supplementary Excel file S11

Supplementary data files

## Data Availability

More detailed breakpoint information and data from the investigated acute leukemia patients can be made available to scientist upon request.
