# Supplementary material for: CRISPR/Cas9-mediated t(4;11) translocation in human hematopoietic stem/precursor cells demonstrates plasticity to differentiate into either the myeloid or lymphoid lineage
Source: Leukemia. 2025 Oct 27;40(1):72–86. doi: 10.1038/s41375-025-02791-4 (PMC12789029; doi:10.1038/s41375-025-02791-4)
Supplement: Supplementary file 1 — Supplementary Figure S1 [file 41375_2025_2791_MOESM1_ESM.docx]

**Supplemental Figure S1**

**
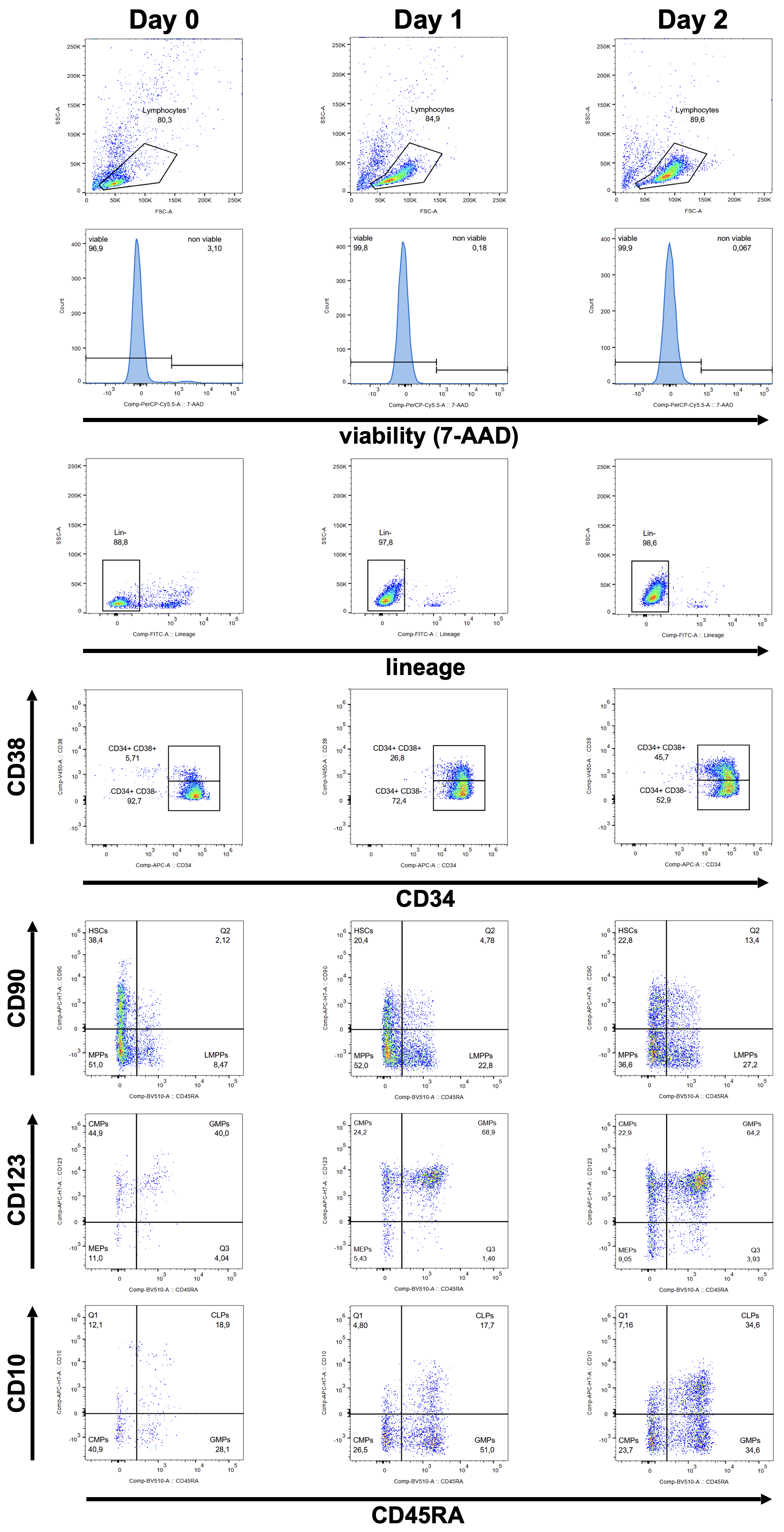
**

**Supplement Figure S1: Proliferation assay and phenotype characterization within culture day 0 to day 3.** Phenotype characterization by flow cytometry. HSCs: Lin^-^ CD34^+^ CD38^-^ CD90^+^ CD45RA^-^; MPPs: Lin^-^ CD34^+^ CD38^-^ CD90^-^ CD45RA^-^; LMPPs: Lin^-^ CD34^+^ CD38^-^ CD90^-^ CD45RA^+^; GMPs: Lin^-^ CD34^+^ CD38^+^ CD123^+^ CD45RA^+^; CMPs: Lin^-^ CD34^+^ CD38^+^ CD123^+^ CD45RA^-^; MEPs: Lin^-^ CD34^+^ CD38^+^ CD123^-^ CD45RA^-^; CLPs: Lin^-^ CD34^+^ CD38^+^ CD10^+^ CD45RA^+^. The figure was generated with FlowJo analysis.
