# Supplementary material for: CRISPR/Cas9-mediated t(4;11) translocation in human hematopoietic stem/precursor cells demonstrates plasticity to differentiate into either the myeloid or lymphoid lineage
Source: Leukemia. 2025 Oct 27;40(1):72–86. doi: 10.1038/s41375-025-02791-4 (PMC12789029; doi:10.1038/s41375-025-02791-4)
Supplement: Supplementary file 13 — Supplementary data files [file 41375_2025_2791_MOESM13_ESM.docx]

**Supplementary data files**

**Supplement Figure S1: Proliferation assay and phenotype characterization within culture day 0 to day 3.** Phenotype characterization by flow cytometry. HSCs: Lin^-^ CD34^+^ CD38^-^ CD90^+^ CD45RA^-^; MPPs: Lin^-^ CD34^+^ CD38^-^ CD90^-^ CD45RA^-^; LMPPs: Lin^-^ CD34^+^ CD38^-^ CD90^-^ CD45RA^+^; GMPs: Lin^-^ CD34^+^ CD38^+^ CD123^+^ CD45RA^+^; CMPs: Lin^-^ CD34^+^ CD38^+^ CD123^+^ CD45RA^-^; MEPs: Lin^-^ CD34^+^ CD38^+^ CD123^-^ CD45RA^-^; CLPs: Lin^-^ CD34^+^ CD38^+^ CD10^+^ CD45RA^+^. The figure was generated with FlowJo analysis.

**Supplemental Excel file S2 – all tpm values:** All transcripts per million (tpm) values of all triplicates were displayed. The mean values were calculated only from triplicates with a minimum of two values ≠ 0. If all means of all samples = 0 those genes were deleted.

**Supplemental Excel file S3 – log2FC and p values:** The calculation of the log2FC values and the corresponding p values for day 14 t(4;11), day 30 t(4;11), day 70 CD19^-^ t(4;11) and day 70 CD19^+^ t(4;11) in comparison with day 14 non-CRISPR/Cas9 edited. Additionally, log2FC values and p values for day 70 CD19^-^ t(4;11) and day 70 CD19^+^ t(4;11) in comparison with day 30 t(4;11) were calculated. If all means of all samples = 0 those genes were deleted. The p values were calculated with a one tailed students t test.

**Supplemental Excel file S4 – Heatmap 1:** Log2FC values from the data comparison of day 14 non-CRISPR/Cas9 edited vs day 14 t(4;11), day 30 t(4;11), day 70 CD19^-^ t(4;11) and day 70 CD19^+^ t(4;11) were used for the heatmap. Only significant p values ≤ 0.05 of sample day 70 CD19^+^ t(4;11) were selected for better lineage comparison. Significant log2FC values ≥ 5 and ≤ -5 were used for the heatmap generation.

**Supplemental Excel file S5 – Heatmap 2:** Log2FC values from the data comparison of day 14 non-CRISPR/Cas9 edited vs day 70 CD19^-^ t(4;11) and day 70 CD19^+^ t(4;11) were used for the heatmap. Only significant p values ≤ 0.05 of sample day 70 CD19^+^ t(4;11) and day 70 CD19^-^ t(4;11) were selected. Equal values were deselected to avoid redundancy.

**Supplemental Excel file S6 – Heatmap 3:** Log2FC values from the data comparison of day 14 non-CRISPR/Cas9 edited vs day 14 t(4;11), day 30 t(4;11), day 70 CD19^-^ t(4;11) and day 70 CD19^+^ t(4;11) were used for the heatmap. Only p values ≤ 0.05 were used if significance was present for day 14 t(4;11), day 30 t(4;11), day 70 CD19^-^ t(4;11) and/or day 70 CD19^+^ t(4;11). Typical leukemia specific genes were selected according to the literature.

**Supplemental Excel file S7a – Volcano 1:** Log2FC values and p values for day 70 CD19^-^ t(4;11) in comparison with day 30 t(4;11) were used for the volcano plot. Only significant p values ≤ 0.05 and genes with mean tpm values ≥ 2 for day 30 t(4;11) and day 70 CD19^-^ t(4;11) were selected to reach a better comparison.

**Supplemental Excel file S7b – Volcano 2:** Log2FC values and p values for day 70 CD19^+^ t(4;11) in comparison with day 30 t(4;11) were used for the volcano plot. Only significant p values ≤ 0.05 and genes with mean tpm values ≥ 2 for day 30 t(4;11) and day 70 CD19^+^ t(4;11) were selected to reach a better comparison.

**Supplemental Excel file S8 – GSEA:** Log2FC values from the data comparison of day 14 non-CRISPR/Cas9 edited vs day 70 CD19^-^ t(4;11) and day 70 CD19^+^ t(4;11) were used for the gene set enrichment analysis (GSEA). Only significant p values ≤ 0.05 and log2FC values ≥ 8 were used for the analysis. The most upregulated genes were analyzed with the Enrichr database on the disease phenotype and cancer cell line similarities. For the disease determination the OMIM Expanded database was used..

**Supplemental Excel file S9 – Comparison to patient data – Heatmap 4a_b:** Up and down regulated genes from t(4;11) infant leukemia patients and t(4;11) non-infant leukemia patients were obtained from Trentin *et al.*.^20^ Infant patients were defined with an age < 1 year. Non-infant patients were defined with an age > 1 year (including children and adults). Up and down regulated genes were defined as followed: upregulated genes: Log2FC > 0; downregulated genes: Log2FC < 0; unchanged genes: Log2FC = 0. All upregulated genes were annotated with a value of 1. All downregulated genes were annotated with a value of -1. All unchanged genes were annotated with a value of 0.

**Supplement Excel file S10 – LSC17 signature – Heatmap 5:** Log2FC values from the data comparison of day 7 non-CRISPR/Cas9 edited vs day 7 t(4;11), day 14 t(4;11), day 30 t(4;11), day 70 CD19- t(4;11) and day 70 CD19+ t(4;11) were used for the heatmap. A selected set of 17 genes from the data purchased by the LSC17 signature were analyzed.^45,46^

**Supplement Excel file S11 – Interesting Target Genes – Heatmap 6:** Log2FC values from the data comparison of day 7 non-CRISPR/Cas9 edited vs day 7 t(4;11), day 14 t(4;11), day 30 t(4;11), day 70 CD19- t(4;11) and day 70 CD19+ t(4;11) were used for the heatmap. Only p values ≤ 0.05 of either day 70 CD19^-^ and/or CD19^+^ were used for heatmap generation.
